# Mixed Criticality Scheduling for Industrial Wireless Sensor Networks

**DOI:** 10.3390/s16091376

**Published:** 2016-08-29

**Authors:** Xi Jin, Changqing Xia, Huiting Xu, Jintao Wang, Peng Zeng

**Affiliations:** 1 Laboratory of Networked Control Systems, Shenyang Institute of Automation, Chinese Academy of Science, Shenyang 110016, China; jinxi@sia.cn (X.J.); xiachangqing@sia.cn (C.X.); wangjintao@sia.cn (J.W.); 2 College of Information Science and Engineering, Northeastern University, Shenyang 110819, China; xuht_neu@hotmail.com; 3 School of Computer and Control Engineering, University of Chinese Academy of Science, Beijing 100049, China

**Keywords:** mixed criticality, industrial wireless sensor networks, scheduling algorithm, scheduling analysis

## Abstract

Wireless sensor networks (WSNs) have been widely used in industrial systems. Their real-time performance and reliability are fundamental to industrial production. Many works have studied the two aspects, but only focus on single criticality WSNs. Mixed criticality requirements exist in many advanced applications in which different data flows have different levels of importance (or criticality). In this paper, first, we propose a scheduling algorithm, which guarantees the real-time performance and reliability requirements of data flows with different levels of criticality. The algorithm supports centralized optimization and adaptive adjustment. It is able to improve both the scheduling performance and flexibility. Then, we provide the schedulability test through rigorous theoretical analysis. We conduct extensive simulations, and the results demonstrate that the proposed scheduling algorithm and analysis significantly outperform existing ones.

## 1. Introduction

Wireless sensor networks (WSNs) make industrial systems low-cost and easy-to-use. Additionally, industrial wireless standards, e.g., WIA-PA (wireless network for industrial automation–process automation) [1], WirelessHART [2,3] and ISA 100.11a (international society of automation) [4], have been developed to promote the popularization of wireless technology. The real-time performance and reliability are essential to industrial systems. Industrial WSNs, as the communication media in industrial systems, must be capable of supporting real-time and reliable communications. The strict requirements on the real-time performance and reliability are different from normal WSNs. Researchers have proposed some scheduling and analyzing methods, e.g., [5,6,7,8,9,10], to improve the two aspects.

**Figure 1:**
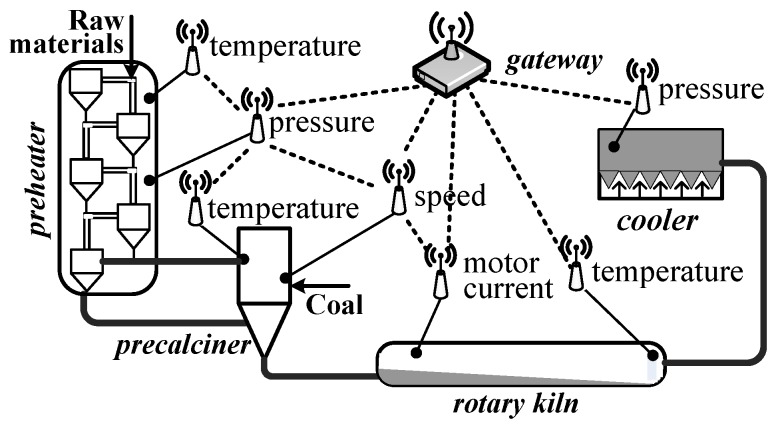
An industrial wireless sensor network in a cement factory.

However, these previous works do not consider the mixed criticality network. Mixed criticality means that different data flows have different levels of importance (or criticality) [11]. For example, Figure 1 shows an industrial WSN for cement manufacturing. The rotary kiln is the most important equipment. If the rotary kiln has some exceptions and its temperature data are lost or miss the deadline, workers cannot take measures in time. This will lead to production inefficiency. By contrast, for the temperature data of pre-heaters, even if they cannot be delivered to the destination within the deadlines, the temperature of materials can be sensed in the pre-calciner. Therefore, the temperature data of the rotary kiln have more importance or higher criticality than those of pre-heaters. Usually, in mixed criticality networks, when the important equipment has an exception, its sensed data have to be quickly and reliably delivered to the control room. This process needs more network resources. In a network, flows with different levels of importance coexist. For resource-constrained WSNs, when resources cannot guarantee the requirements of all levels, the data flows that belong to less importance levels should be discarded. The discard strategy is applied in almost all of the mixed criticality systems [12]. Thus, the flows are dynamic in mixed criticality networks. To guarantee the real-time performance and reliability, previous works on industrial single criticality WSNs apply totally centralized methods to manage networks, e.g., [5,13,14]. However, the totally centralized methods have difficulty coping with dynamic flows.

Intuitively, two types of methods can be used to schedule data flows in mixed criticality WSNs. The first type is to schedule flows based on criticality monotonic priorities. The criticality monotonic scheduling assigns the higher priority to the important flows and schedules them first. This method does not cope with the dynamism and transmits important flows as soon as possible. In this method, the criticality is considered as the temporality. However, actually, they are not equivalent. The criticality means the importance, while the temporality means the urgency. The temperature data of the rotary kiln are the most important, but the pressure data of pre-heaters are more urgent than those, since the change of pressure is the most frequent. If the temperature data are transmitted first, the urgent pressure data will miss their deadline, even though there are idle network resources after their deadlines. Thus, the criticality monotonic scheduling algorithm cannot utilize resources efficiently and is not suitable for mixed criticality systems. This has also been demonstrated in [15]. The second type is to use the algorithms that have been proposed for previous mixed criticality systems, such as uniprocessor/multiprocessor systems [16,17,18] and networks [19,20,21], to solve our problem. However, industrial WSNs are different from the previous systems. To guarantee the strict requirements on the real-time performance and reliability, the main problem to be solved is how to avoid the collision and interference between parallel data flows. Mixed criticality uniprocessor/mulitprocessor systems only consider independent processors and do not have the interference between parallel tasks. Mixed criticality wired networks and IEEE 802.11-based wireless networks are based on CSMA (carrier sense multiple access) protocols, which are unacceptable by industrial WSNs due to the unpredictability (we give more clarifications on the differences between our system and others in Section 2). Therefore, previous algorithms cannot be used without modification in mixed criticality industrial WSNs. In this paper, we present a holistic scheduling solution to guarantee the real-time and reliability requirements of data flows in resource-constrained industrial WSNs. Our scheduling method is implemented in the application layer. According to the generated schedules, each network node transmits or receives packets in the MAC (medium access control) layer. The scheduling method of the application layer manages all data flows based on global information. Thus, it can get the optimized solution. Some MAC protocols, e.g., [22,23], are proposed to improve the real-time performance and reliability for industrial wireless sensor networks. They are flexible and scalable, but are difficult to optimize globally because they transmit packets based on local information. We list our contributions as follows.

First, we introduce the concept of mixed criticality into resource-constrained industrial WSNs. The mixed criticality concept distinguishes important data flows from less important data flows. It provides a new vision for resource-constrained networks to meet the high performance requirement of important flows.

Second, we propose a scheduling algorithm for the mixed criticality network. The scheduling algorithm not only implements the optimized global management for all flows, but also reserves network resources for dynamic adjustments to enhance the real-time performance and reliability of important flows. It makes a trade-off between the scheduling performance and the flexibility. Performance evaluations demonstrate that the proposed scheduling algorithm outperforms existing ones.

Third, we present a schedulability analysis for the proposed scheduling algorithm. We analyze end-to-end delay for flows and determine whether they are all schedulable. Simulation results show that our schedulability analysis is more effective than existing ones.

The rest of the paper is organized as follows. Section 2 introduces related works. Section 3 presents our system model. Section 4 formulates our problem as a satisfiability modulo theories instance. Section 5 proposes a heuristic scheduling algorithm for mixed criticality WSNs. Section 6 presents the delay analysis and the schedulability test. Section 7 shows simulation results. Section 8 concludes this paper.

## 2. Related Works

Scheduling algorithms and schedulability analysis methods have been widely studied in single criticality WSNs. The work in [13] proposes a real-time scheduling algorithm and analyzes the schedulability for industrial WSNs with linear topology, and the work in [24] presents similar methods for binary-tree networks. Based on the above two works, the work in [25] takes the impact of packet copying into account to enhance the channel utilization, and the work in [14] supports spatial reuse to improve the schedulability. For mesh topology networks, the authors of [5,6,26] propose a scheduling analysis, a fixed priority scheduling algorithm and two dynamic priority scheduling algorithms to meet the real-time requirement of industrial applications. However, these previous works do not consider the mixed criticality requirement.

Mixed criticality is first proposed in uniprocessor systems [16,27]. Then, it is introduced to multiprocessor systems [17,18], controller area networks [19], network-on-chips [28,29], wired networks [20,30] and IEEE 802.11-based wireless networks [21]. Uniprocessor systems [16,27] and controller area networks [19] do not support parallel tasks (or data flows); while the serial mode is unsuitable for industrial WSNs. The works in [17,18] focus on homogeneous multiprocessor systems. As there is no interference between executing tasks, they do not need to consider how to avoid the interference. However, in industrial WSNs, the interference must be avoided. Network-on-chips use wormhole switching. For one data flow, the network-on-chip has to provide all nodes that the data flow uses simultaneously [28,29], whereas the industrial WSN only provides two nodes for one hop. Wired networks [20,30] and IEEE 802.11-based wireless networks [21] are based on the CSMA protocol, which is unacceptable by reliable industrial systems. Therefore, the previous system models are different from industrial WSNs.

In WSNs, some works about critical data flows have been proposed. The IEEE 802.15.4 standard, which is widely used by WSNs, supports a hybrid TDMA/CSMA MAC protocol. In the TDMA frame, the guaranteed time slots (GTSs) can be assigned for transmitting critical data flows. For the hybrid protocol, the works in [31,32] propose time slot assignment algorithms to improve the performance of critical data flows. However, due to the unpredictability of the CSMA protocol, industrial WSNs cannot apply the hybrid protocol. The work in [22] considers a pure TDMA protocol. It proposes the PriorityMAC protocol, which is a distributed method and allows critical data flows to be transmitted as soon as possible. The PriorityMAC protocol assumes that the most important data flows are urgent; while in the mixed criticality industrial WSN, the importance and the urgency are independent of each other. We have researched mixed criticality industrial WSNs in our previous work [33]. In that work, the network adopts a totally centralized management, and the end-to-end delay is calculated. However, this strict centralized management is not flexible enough, and all exceptions have to be submitted to the centralized manager and wait to be processed. In this paper, our proposed mixed criticality network supports the dynamic adaptive strategy. The classical RM (rate-monotonic) scheduling policy, which first schedules the flow with a shorter period, is a basic scheduling strategy and has been widely used in industrial WSNs [1,2]. We will extend the classical RM policy and allow the important data flows to preempt the resources assigned to less important data flows. Then, we will analyze the schedulability of our extended RM policy.

## 3. System Model

Industrial WSNs must support the strict requirements on real-time performance and reliability. Therefore, we consider an industrial WSN as follows. It consists of a gateway and some sensor devices (as shown in Figure 1). We use the node set $N=\{n_{1},n_{2},...\}$ to denote these nodes. The physical layer of our industrial WSNs is specified by the IEEE 802.15.4 protocol. It supports 16 non-overlapping channels. However, due to external interference, not all of them can be accessed all of the time. We denote the number of available channels as *M* (1≤M≤16). Our network serves the flow set $F=\{f_{1},f_{2},...\}$. Each element $f_{i}$ is characterized by $<T_{i},\Pi_{i},\chi_{i}>$. Each flow $f_{i}$ periodically generates a packet at its period $T_{i}$ and then sends it to the destination via the routing path $\Pi_{i}$. The relative deadline of each packet is equal to the period $T_{i}$, i.e., a packet is released at the time *t*, and it must be delivered to its destination before the time $\left(t+T_{i}+1\right)$. In industrial wireless protocol, e.g., [1,2], periods conform to the expression:

(1) $$b\times 2^{a}$$

where *a* is an integer value and *b* is the unit-period.

To keep consistent with related works on mixed criticality systems, our network also supports two criticality levels, L-crit (low criticality) and H-crit (high criticality). The dual-criticality model can be easily extended to the multi-criticality model. If the flow $f_{i}$ is important, its criticality level $\chi_{i}$ is denoted as *H*. Otherwise, its criticality level $\chi_{i}$ is *L*. When the system is running in the normal mode without any exception, all flows are delivered to their destinations within deadlines. If an important equipment has an exception, the corresponding data must be submitted frequently and via two paths to avoid faults on a single path. Thus, in our system model, the H-crit flows have two parameter sets: the L-crit parameters $<T_{i}\left(L\right),\Pi_{i}\left(L\right)>$ in the normal mode; the H-crit parameters $<T_{i}\left(H\right),\Pi_{i}\left(H\right)>$ in the exception mode and $T_{i}\left(H\right)\leq T_{i}\left(L\right)$. $\Pi_{i}\left(L\right)$ is a path that is used by the H-crit flow in the normal mode. $\Pi_{i}\left(H\right)$ contains two paths that are used by the H-crit flow in the exception mode, and the two paths transmit the same packet to improve the reliability. In order to clearly distinguish these paths, they are denoted as $\Pi_{i}\left(L\right)=\left\{\pi_{i}^{*}\right\}$ and $\Pi_{i}\left(H\right)=\left\{\pi_{i}^{\prime },\pi_{i}^{\prime \prime }\right\}$. The path $\pi_{i}^{*}$ (and $\pi_{i}^{\prime }$, $\pi_{i}^{\prime \prime }$) is the set of links from the source to the destination. In this paper, we do not consider how to select routing paths. We assume all paths have been given before generating schedules. The dynamism this paper addresses refers to using different parameters in different modes. Transmitting a packet through the *j*-th link of the path $\pi_{i}^{*}$ (or $\pi_{i}^{\prime }$, $\pi_{i}^{\prime \prime }$) is called the transmission $\tau_{ij}^{*}$ (and $\tau_{ij}^{\prime }$, $\tau_{ij}^{\prime \prime }$). Each transmission has two attributes $<n_{\alpha },n_{\beta }>$, which denote the transmission’s source and destination, respectively. As the constrained-resources must provide enough services to H-crit flows, the L-crit flows cannot be transmitted when exceptions happen. Therefore, L-crit flows only have a parameter set $<T_{i}\left(L\right),\Pi_{i}\left(L\right)>$.

**Figure 2:**
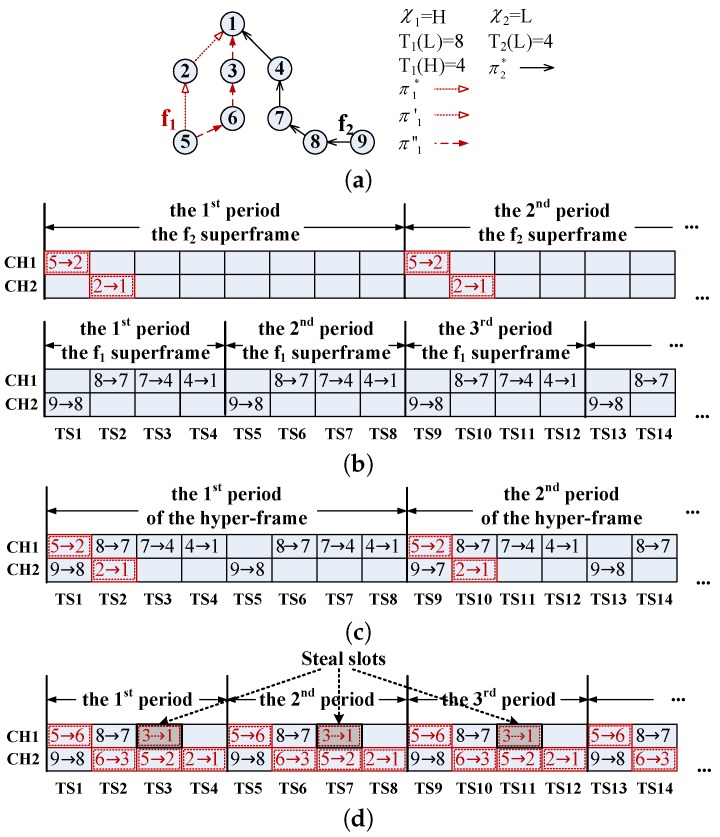
Graph routing and superframe. (**a**) A network; (**b**) superframes with different periods; (**c**) a hyper-frame; (**d**) the flow $f_{2}$ steal slots from the flow $f_{1}$.

To improve the reliability of industrial networks, we adopt the TDMA scheme in the MAC layer. The network manager, which is connected to the gateway, assigns a time slot and a channel offset to each transmission. A transmission only is scheduled at the given time slot and on the given channel offset. Packets are generated periodically, and the schedules of corresponding transmissions have the same period. The schedules with the same period are organized within a superframe [2]. Transmitting a packet from the source to the destination has to be done in a superframe. Thus, superframes repeat themselves periodically, and then, flows can be transmitted successfully. Figure 2a shows a simple network, which contains two flows $f_{1}$ and $f_{2}$. When the system is in normal mode, the flows use their L-crit parameters. Their periods are eight time slots and four time slots, and their paths are $\left\{e_{52},e_{21}\right\}$ and $\left\{e_{98},e_{87},e_{74},e_{41}\right\}$, where $e_{ij}$ denotes the link from the node $n_{i}$ to the node $n_{j}$. Figure 2b shows their superframes with different periods. CH and TS denote channel offset and time slot.

Two types of improper schedules will lead to transmission interference, which seriously affects the network reliability. The first type, called node interference, is that more than one transmission uses the same node at the same time slot. Each node is only equipped with one transmitter. Therefore, one node cannot serve more than one transmission at the same time. The second type is called scheduling interference, which means that more than one transmission is scheduled at the same time slot and on the same channel. These overlapping transmissions cannot be separated. To avoid transmission interference between different superframes, we consider all superframes as a hyper-frame whose period is the lowest common multiple of all superframes. According to the period’s Expression (1), the hyper-period $T=LCM\left(T_{1},T_{2},...\right)=\max_{\forall f_{i}\in F}\left\{T_{i}\right\}$. Figure 2c shows the hyper-frame of the simple example. We only consider how to schedule flows in the first hyper-period, since after that, all schedules are repeated periodically. The network manager generates all schedules under two situations: Situation 1: when the network is deployed; and Situation 2: when the deployment is changed. Due to the requirement of industrial applications being fixed, the deployment is not often changed. Thus, the schedules may be generated several times, but not frequently. According to this schedule information, it obtains the working modes of each node at every time slot and then delivers them to the corresponding nodes. For the schedules in Figure 2c, from TS1 to TS4, working modes of the node $n_{2}$ are {receive, send, idle, idle}.

When a node intends to send a transmission of L-crit flows, it waits for a constant time and then listens whether its channel is used. If the channel is used by H-crit flows, the node discards its transmission. Otherwise, the node sends the transmission. Note that although the node uses the carrier sense technique to determine whether an L-crit transmission is discarded or not, it is different from the CSMA scheme. Since for L-crit flows, the node performs carrier sense within time slots of the TDMA frame, if the L-crit transmission is not discarded, it is also scheduled based on the TDMA scheme. When a node intends to send a transmission of H-crit flows, it immediately sends it at the beginning of the assigned time slot. The scheduling algorithm assigns the proper time slot and channel for each transmission and prevents H-crit transmissions from interfering with other H-crit transmissions. Therefore, H-crit transmissions are sent directly without checking the channel. In this way, the H-crit flow can steal slots from L-crit flows when it needs more resources to cope with exceptions [34]. Note that the H-crit flow using H-crit parameters is not permitted to steal slots that are used by any other H-crit flows even if these H-crit flows are using L-crit parameters. Figure 2d shows an example of mixed criticality schedules. The period of the H-crit flow $f_{1}$ is changed from eight to four, and the new path $\left\{e_{56},e_{63},e_{31}\right\}$ begins to be used. In this case, there are not enough time slots. The H-crit transmission 3→1 (the gray block) steals the resource of the L-crit transmission 7→4. Based on the stealing strategy, the dynamic adjustment can be supported.

The schedulable flow set is defined as follows. When the system is in the normal mode, the flow set is schedulable if all flows characterized by L-crit parameters can hit their deadlines. When there are exceptions in the system, the flow set is schedulable if all H-crit flows can hit their deadlines no matter which parameters they are using.

## 4. Mixed Criticality Scheduling Problem Statement

Based on the above system model, we describe the mixed criticality scheduling problem as follows. Given the network and the flow set *F*, our objective is to schedule transmissions in the time slot and channel dimensions, such that the flow set is schedulable.

To explain the problem more clearly, we formulate the problem as a satisfiability modulo theories (SMT) specification. The transmission $\tau_{ij}^{*}$ (and $\tau_{ij}^{\prime }$, $\tau_{ij}^{\prime \prime }$) is assigned the $s_{ij}^{*}$-th (and $s_{ij}^{\prime }$-th, $s_{ij}^{\prime \prime }$-th) time slot and the $r_{ij}^{*}$-th (and $r_{ij}^{\prime }$-th, $r_{ij}^{\prime \prime }$-th) channel offset. Note that a transmission is scheduled periodically. Therefore, the transmission uses all of the time slots $s_{ij}+g\cdot T_{i}$ $\left(\forall g\in \left[0,\frac{T}{T_{i}}\right)\right)$ in a hyper-frame. These assignments must respect the following constraints.

(a) Channel offset constraint:
  $$\forall f_{i},\forall j\in \left[1,\right|\pi_{i}^{*}\left|\right],1\leq r_{ij}^{*}\leq M$$
  For each transmission, its assigned channel offset must be in *M* available channels. This expression is for transmissions in the path $\pi_{i}^{*}$. Other transmissions $\tau_{ij}^{\prime }$ and $\tau_{ij}^{\prime \prime }$ in paths $\pi_{i}^{\prime }$ and $\pi_{i}^{\prime \prime }$ have the same constraint, and we omit them for simplicity.
(b) Releasing sequence constraint:
  $$\forall f_{i},\forall j\in \left[1,\right|\pi_{i}^{*}\left|-1\right],s_{i,j}^{*}<s_{i,j+1}^{*}$$
  In a routing path, the transmission $\tau_{i,j+1}$ is released after the transmission $\tau_{i,j}$ is scheduled. We still omit paths $\pi_{i}^{\prime }$ and $\pi_{i}^{\prime \prime }$.
(c) Real-time constraint:
  $$\forall f_{i},1\leq s_{i,|\pi_{i}^{*}|}^{*}\leq T_{i}\left(L\right)$$
  All transmissions cannot miss deadlines. Likewise, $s_{i,|\pi_{i}^{\prime }|}^{\prime }$ and $s_{i,|\pi_{i}^{\prime \prime }|}^{\prime \prime }$ have the same constraint.
(d) Interference constraint: Assigning resources to transmissions must prevent the happening of node interference and scheduling interference. We use $\delta (\tau_{a},\tau_{b})$ to denote whether there exists interference between $\tau_{a}$ and $\tau_{b}$,
  $$\delta \left(\tau_{a},\tau_{b}\right)=\left(\tau_{a}\cap \tau_{b}=\emptyset \right)?\left(\eta \left(s_{a},s_{b}\right)\wedge \left(r_{a}=r_{b}\right)\right):\eta \left(s_{a},s_{b}\right)$$
  where $\eta \left(s_{a},s_{b}\right)=\underset{\forall h\in \left[0,\frac{T}{T_{a}}\right),\forall k\in \left[0,\frac{T}{T_{b}}\right)}{⋁}\left(s_{a}+h\cdot T_{a}=s_{b}+k\cdot T_{b}\right)$ means whether the assigned time slots of $\tau_{a}$ and $\tau_{b}$ overlap each other. If the two transmissions do not use the same node, i.e., $\tau_{a}\cap \tau_{b}=\emptyset$, then they can be scheduled at different time slots or on the different channel offsets. Otherwise, there exists node interference, and they cannot be scheduled at the same time slot. The transmissions of the H-crit flow $f_{i}$ are classified into three sets $\Gamma_{i}^{*}=\{\tau_{ij}^{*}\left|\forall j\in [1,\right|\pi_{i}^{*}\left|]\right\}$, $\Gamma_{i}^{\prime }=\{\tau_{ij}^{\prime }\left|\forall j\in [1,\right|\pi_{i}^{\prime }\left|]\right\}$ and $\Gamma_{i}^{\prime \prime }=\{\tau_{ij}^{\prime \prime }\left|\forall j\in [1,\right|\pi_{i}^{\prime \prime }\left|]\right\}$. For the L-crit flow $f_{i}$, $\Gamma_{i}^{\prime }=\Gamma_{i}^{\prime \prime }=\emptyset$, and then, $\forall f_{i}\in F,\Gamma_{i}=\Gamma_{i}^{*}\cup \Gamma_{i}^{\prime }\cup \Gamma_{i}^{\prime \prime }$. Thus, the interference constraint in the normal mode and exception mode are as follows.
  (d.1) Normal mode:
    $$\forall \tau_{a},\tau_{b}\in \underset{\forall f_{i}\in F}{⋁}\Gamma_{i}^{*},\delta \left(\tau_{a},\tau_{b}\right)=0$$
  (d.2) Exception mode:
    $$\forall f_{i},f_{g}\in F,\chi_{i}=\chi_{g}=H,\forall \tau_{a}\in \Gamma_{i},\forall \tau_{b}\in \Gamma_{g},\delta \left(\tau_{a},\tau_{b}\right)=0$$

The mixed criticality scheduling problem is NP-hard [11]. Our SMT specification can be solved by some solvers, such as Z3 [35] and Yices [36]. These solvers can find satisfying assignments for quite many problems, and their solutions have been the excellent standard to evaluate the effectiveness of other methods [37]. However, the running time may be unacceptable for complex networks and flow sets. Therefore, we propose a heuristic scheduling algorithm in Section 5 to solve the problem.

## 5. Scheduling Algorithm

In this section, we first introduce how to schedule transmissions, and then, based on these schedules, we determine working modes of each node at every time slot.

### 5.1. A Slot-Stealing Scheduling Algorithm

We propose a slot-stealing scheduling algorithm based on RM (StealRM). The proposed StealRM optimizes the solution according to the global information and permits transmissions to share the same resource when the transmissions have different levels of criticality. Hence, the schedules can be adaptively adjusted based on the requirements of H-crit flows.

The proposed StealRM is shown in Algorithm 1. Each flow is assigned as the RM priority. If two flows have the same RM priority, the flow with the smaller ID has the higher priority. The transmission’s priority is equal to its flow’s priority. The set *R* contains all of schedulable transmissions (Lines 1 and 17), and the set $R^{\prime }$ denotes released transmissions at the current time slot (Line 3). At every time slot *t*, we first sort elements of $R^{\prime }$ according to the decreasing order of priorities, and $\tau_{1}$ in the set $R^{\prime }$ has the highest priority (Line 4). Then, for each transmission $\tau_{a}$ in the set $R^{\prime }$, we check whether it can be scheduled at the current time slot without any interference (lines between 7 and 21). Let $F(\tau_{a})$ denote the flow that the transmission $\tau_{a}$ belongs to (Line 6). The set $Y_{t}^{HL}$ contains the transmissions that have been scheduled at the time slot *t* and belong to H-crit flows with L-crit parameters. The sets $Y_{t}^{H}$ and $Y_{t}^{L}$ correspond to those in H-crit flows with H-crit parameters and L-crit flows, respectively. The transmissions in the set $Y^{\prime }$ and the transmission $\tau_{a}$ cannot steal slots from each other. According to the criticality level of $\tau_{a}$, the set $Y^{\prime }$ is assigned different transmissions (lines between 7 and 12). If the transmission $\tau_{a}$ belongs to an H-crit flow with H-crit parameters, then it cannot steal slots from other H-crit transmissions (lines between 7 and 8). $Y^{H}$ and $Y^{HL}$ may contain the transmissions belonging to the same flow with $\tau_{a}$. These transmissions do not interfere the scheduling of $\tau_{a}$. Thus, the set $\left\{\forall \tau_{ig}^{*}\right\}$ needs to be excluded from $Y^{H}$ and $Y^{HL}$ (Line 8). Similarly, if the transmission $\tau_{a}$ belongs to an H-crit flow with L-crit parameters, then it cannot steal slots from any other transmissions (lines between 9 and 10). If the transmission $\tau_{a}$ belongs to an L-crit flow, then its slots cannot be stolen by L-crit flows and H-crit flows with L-crit parameters (lines between 11 and 12). When there is no node interference between $\tau_{a}$ and $Y^{\prime }$ and at least one channel is idle (Line 13), the transmission $\tau_{a}$ can be scheduled at this current time slot. $\Theta (Y^{\prime })$ denotes the channels that have been used by $Y^{\prime }$. However, if the current time slot has exceeded its deadline, the flow set is unschedulable (Lines 14 and 15). Otherwise, the time slot and channel offset of the transmission $\tau_{a}$ are assigned (Line 16), and the schedulable transmission set *R* and the scheduled transmission set $Y_{t}^{H}$ ($Y_{t}^{L}$ and $Y_{t}^{HL}$) are updated (lines between 17 and 23).
**Algorithm 1** StealRM.**Require:** the flow set *F***Ensure:** the scheduling results $\forall s_{a}$ and $\forall r_{a}$ 1: the schedulable transmission set $R\leftarrow \{\tau_{i1}^{*},\tau_{i1}^{\prime },\tau_{i1}^{\prime \prime }|\forall f_{i}\in F\}$; 2: **for**
∀t∈[1,T]**do** 3:  $R^{\prime }\leftarrow R$; 4:  sort $R^{\prime }$ according to the decreasing order of priorities; 5:  **for** each *a* from 1 to $|R^{\prime }|$
**do** 6:   $i\leftarrow F(\tau_{a})$ 7:   **if**
$\chi_{i}==H$ and $\tau_{a}\in \Gamma_{i}^{\prime }\cup \Gamma_{i}^{\prime \prime }$
**then** 8:    $Y^{\prime }\leftarrow \underset{\forall h\in [0,\frac{T}{T_{i}\left(H\right)})}{\cup }\left(Y_{t+T_{i}\left(H\right)\times h}^{H}\cup Y_{t+T_{i}\left(H\right)\times h}^{HL}\right)-\left\{\forall \tau_{ig}^{*}\right\}$; 9:   **else**
**if**
$\chi_{i}==H$ and $\tau_{a}\in \Gamma_{i}^{*}$
**then**10:    $Y^{\prime }\leftarrow \left(\underset{\forall h\in [0,\frac{T}{T_{i}\left(L\right)})}{\cup }Y_{t+T_{i}\left(L\right)\times h}^{L}\right)\cup \left(\underset{\forall h\in [0,\frac{T}{T_{i}\left(H\right)})}{\cup }\left(Y_{t+T_{i}\left(H\right)\times h}^{H}\cup Y_{t+T_{i}\left(H\right)\times h}^{HL}\right)\right)-\left\{\forall \tau_{ig}^{\prime },\tau_{ig}^{\prime \prime }\right\}$;11:   **else**12:    $Y^{\prime }\leftarrow \left(\underset{\forall h\in [0,\frac{T}{T_{i}\left(L\right)})}{\cup }Y_{t+T_{i}\left(L\right)\times h}^{L}\right)\cup \left(\underset{\forall h\in [0,\frac{T}{T_{i}\left(H\right)})}{\cup }Y_{t+T_{i}\left(H\right)\times h}^{HL}\right)$;13:   **if**
$\underset{\forall \tau_{b}\in Y^{\prime }}{⋀}\left(\tau_{a}\cap \tau_{b}\neq \emptyset \right)$ and $|\Theta (Y^{\prime })|<M$
**then**14:    **if**
*t* exceeds the deadline of $f_{i}$
**then**15:     **return** unschedulable;16:    $s_{a}\leftarrow t$; $r_{a}\leftarrow$ a random channel that is not in $\Theta (Y^{\prime })$;17:    $R\leftarrow R-\{\tau_{a}\}+$ the next transmission of $\tau_{a}$;18:    **if**
$\chi_{i}==H$ and $\tau_{a}\in \Gamma_{i}^{\prime }\cup \Gamma_{i}^{\prime \prime }$
**then**19:     $\forall h\in \left[0,\frac{T}{T_{i}\left(H\right)}\right),Y_{t+T_{i}\left(H\right)\times h}^{H}\leftarrow Y_{t+T_{i}\left(H\right)\times h}^{H}+\left\{\tau_{a}\right\}$;20:    **else**
**if**
$\chi_{i}==H$ and $\tau_{a}\in \Gamma_{i}^{*}$
**then**21:     $\forall h\in \left[0,\frac{T}{T_{i}\left(L\right)}\right),Y_{t+T_{i}\left(L\right)\times h}^{HL}\leftarrow Y_{t+T_{i}\left(L\right)\times h}^{HL}+\left\{\tau_{a}\right\}$;22:    **else**23:     $\forall h\in \left[0,\frac{T}{T_{i}\left(L\right)}\right),Y_{t+T_{i}\left(L\right)\times h}^{L}\leftarrow Y_{t+T_{i}\left(L\right)\times h}^{L}+\left\{\tau_{a}\right\}$;24: **return**
$\forall s_{a}$ and $\forall r_{a}$;

The number of iterations of the for loop in Line 2 and the for loop in Line 5 is O(|T|) and O(|Γ|), respectively. The complexity of Line 4, Line 13 and Line 19 is O(|Γ|log|Γ|), O(|Γ|) and $O(\frac{T}{T_{min}})$, respectively. Therefore, the time complexity of Algorithm 1 is $O(|T||\Gamma {|}^{2}\frac{T}{T_{min}})$.

### 5.2. Node Working Mode

Nodes have three working modes, including transmit mode (S), receive mode (R) and idle mode. We use $w_{\alpha ,t}^{H}=<S\;\text{(or}\;R),r_{a}>$ to denote that at the time slot *t*, the node $n_{\alpha }$ transmits (or receives) H-crit flows on the channel $r_{a}$. Similarly, $w_{\alpha ,t}^{L}$ denotes that the node $n_{\alpha }$ serves L-crit flows. Algorithm 2 determines the working mode for each node. For each transmission, we have assigned a time slot and a channel offset in Algorithm 1. According to the assignments, the working modes of the sender node and receiver node of the transmission can be obtained (lines between 4 and 9). The time complexity of Algorithm 2 is $O(|\Gamma |\frac{T}{T_{min}})$.
**Algorithm 2** Working mode.**Require:** the scheduling results $\forall s_{a}$ and $\forall r_{a}$**Ensure:** all $w_{*,*}^{L}$ and $w_{*,*}^{H}$ 1: all $w_{*,*}^{L}$ and $w_{*,*}^{H}$ are initiated as idle mode; 2: **for**
$\forall \tau_{a}\in \Gamma$
**do** 3:  $i\leftarrow F(\tau_{a})$; $<n_{\alpha },n_{\beta }>$ are the sender and receiver of $\tau_{a}$; 4:  **if**
$\chi_{i}==H$
**then** 5:   $\forall h\in \left[0,\frac{T}{T_{i}\left(H\right)}\right),w_{\alpha ,s_{a}+T_{i}\left(H\right)\times h}^{H}\leftarrow <S,r_{a}>$; 6:   $\forall h\in \left[0,\frac{T}{T_{i}\left(H\right)}\right),w_{\beta ,s_{a}+T_{i}\left(H\right)\times h}^{H}\leftarrow <R,r_{a}>$; 7:  **else** 8:   $\forall h\in \left[0,\frac{T}{T_{i}\left(L\right)}\right),w_{\alpha ,s_{a}+T_{i}\left(L\right)\times h}^{L}\leftarrow <S,r_{a}>$; 9:   $\forall h\in \left[0,\frac{T}{T_{i}\left(L\right)}\right),w_{\beta ,s_{a}+T_{i}\left(L\right)\times h}^{L}\leftarrow <R,r_{a}>$;10: **return** all $w_{*,*}^{L}$ and $w_{*,*}^{H}$;

Note that a node may serve two flows at the same time slot, but the two flows must have different criticality levels. Otherwise, node interference occurs. At the beginning of the time slot *t*, the node works in mode $w_{\alpha ,t}^{H}$. Then, in a constant time, if it needs to send an H-crit flow or has received a flow, it continues working as the same mode at this time slot. Otherwise, it works in mode $w_{\alpha ,t}^{L}$. However, when its mode $w_{\alpha ,t}^{L}$ is S, it must determine whether the assigned channel is clear or not before it sends the flow. If the channel has been occupied by H-crit flows, the flow has to be discarded. The switch time between different modes is very short compared with a time slot. For example, the switch time of the transceiver CC2420 is just 200 μs, while a time slot is 10 ms. Generally, at a time slot, most nodes only serve one flow or are idle, while only a few nodes serve two flows.

## 6. Scheduling Analysis

In this section, we analyze the worst case end-to-end delay for each flow and use the delay to test the schedulability of the flow set. If the worst case delay of all flows does not exceed deadlines, the flow set is schedulable. For the sake of simplicity, we first explain how to compute the worst case delay in single-criticality networks (in Section 6.1) and then extend it to mixed criticality networks (in Section 6.2).

### 6.1. Analyzing Method for Single-Criticality Networks

Besides transmitting time, the end-to-end delay is introduced by the interference from higher priority flows. Therefore, in Section 6.1.1, we present the analyzing method of the total interference. In Section 6.1.2, we distinguish the different types of interference and compute the worst case delay.

#### 6.1.1. Total Interference

During the time interval between the release and completion of the flow $f_{k}$, all of the active transmissions that belong to the higher priority flows may have node interference or scheduling interference to the flow $f_{k}$. Therefore, in the worst case, the total interference is equal to the number of those higher-priority transmissions. The method of computing the workload in a period has been proposed in multiprocessor systems [38]. The mapping between the multiprocessor system model and the network model has been explained in the work [6], in which a channel corresponds to a processor and a flow is scheduled as a task. Therefore, we propose our analyzing method based on the work [38], which is the start-of-the-art analysis for multiprocessor systems. To make our paper self-contained, we first simply introduce the method of multiprocessor systems and then present our method.

For the simplicity of expression, the multiprocessor system uses the same notations as our network model. For multiprocessor systems, the calculation of the worst case delay of the task $f_{k}$ is based on the level-*k* busy period (as shown in Definition 1).

**Definition 1.**  *Level-k busy period for multiprocessor systems: The level-k busy period is the time interval $\left[t_{0},t_{k}\right)$, in which $t_{k}$ is the finish time of the task $f_{k}$, and $t_{0}$ satisfies the following conditions:*

*1.*  $t_{0}<t_{r}$ where $t_{r}$ is the release time of the task $f_{k}$.
*2.*  $\forall t\in [t_{0},t_{r}]$, at the time t, all processors are occupied by higher-priority tasks.
*3.*  $\forall t<t_{0},\exists t^{\prime }\in \left[t,t_{0}\right]$, at the time $t^{\prime }$, at least one processor is occupied by lower-priority tasks.

If there is no $t_{0}$ that satisfies all conditions, then $t_{0}=t_{r}$.

The level-*k* busy period is determined by the workload of all higher-priority tasks. The set $\bar{P}\left(f_{k}\right)$ contains the tasks with higher priority than the task $f_{k}$. If the task $f_{i}$ ($f_{i}\in \bar{P}\left(f_{k}\right)$) has a job that is released earlier than the level-*k* busy period and its deadline is in the busy period, then the task $f_{i}$ has the carry-in workload in the level-*k* busy period. Otherwise, the task has no carry-in workload. The two types of workloads are presented as follows, and the length of the level-*k* busy period is *x*.

(1) In the level-*k* busy period, if the task $f_{i}$ has no carry-in workload, the upper bound of its workload is:
  $$W_{k}^{NC}\left(f_{i},x\right)=\left\lfloor \frac{x}{T_{i}}\right\rfloor \cdot c_{i}+\min\left\{x\;\mathrm{mod}\;T_{i},c_{i}\right\}$$
  where $c_{i}$ is the execution time of the task $f_{i}$.
(2) If the task $f_{i}$ has the carry-in workload, the upper bound of its workload is:
  $$W_{k}^{CI}\left(f_{i},x\right)=\left\lfloor \frac{\max\{x-c_{i},0\}}{T_{i}}\right\rfloor \cdot c_{i}+c_{i}+\alpha$$
  where $\alpha =\min\{\max\left\{\max\left\{x-c_{i},0\right\}-\left(T_{i}-D_{i}\right),0\right\},c_{i}-1\}$ and $D_{i}$ is the worst case delay of the task $f_{i}$.

Based on the upper bounds of workload, two types of interference of the task $f_{i}$ to the task $f_{k}$ are as follows:

$$I_{k}^{NC}\left(f_{i},x\right)=\min\left\{\max\left\{W_{k}^{NC}\left(f_{i},x\right),0\right\},x-c_{k}+1\right\}$$

$$I_{k}^{CI}\left(f_{i},x\right)=\min\left\{\max\left\{W_{k}^{CI}\left(f_{i},x\right),0\right\},x-c_{k}+1\right\}$$

Therefore, the total interference suffered by the task $f_{k}$ is:

$$\Omega_{k}\left(x,{\bar{P}}^{NC}\left(f_{k}\right),{\bar{P}}^{CI}\left(f_{k}\right)\right)=\sum_{\forall f_{i}\in {\bar{P}}^{NC}\left(f_{k}\right)}I_{k}^{NC}\left(f_{i},x\right)+\sum_{\forall f_{i}\in {\bar{P}}^{CI}\left(f_{k}\right)}I_{k}^{CI}\left(f_{i},x\right)$$

where ${\bar{P}}^{NC}\left(f_{k}\right)$ and ${\bar{P}}^{CI}\left(f_{k}\right)$ denote the set of tasks without carry-in workload and the set of tasks with carry-in workload, respectively. In a busy period, at most M-1 higher-priority tasks have carry-in workload. Therefore, the set ${\bar{P}}^{CI}$ contains M-1 tasks that have maximal values of $I_{k}^{CI}\left(f_{i},x\right)-I_{k}^{NC}\left(f_{i},x\right)$. Other tasks are in the set ${\bar{P}}^{NC}$.

In the following, we propose our analyzing method. Industrial WSNs apply strict periodic schedules based on superframes, which can reduce system complexity and run time overhead. While in multiprocessor systems and previous works about WSNs, schedules are variable, i.e., the assigned time slots to a task (or a flow) are non-periodic, so our workload bounds are not the same as previous ones. Our workload bounds are computed with Theorem 1. Definition 2 defines the level-*k* busy period in the network.

**Definition 2.**  *Level-k busy period for networks: The level-k busy period is the time interval $\left[t_{0},t_{k}\right)$, in which $t_{k}$ is the finish time of the flow $f_{k}$ and $t_{0}$ satisfies the following conditions:*

*1.*  $t_{0}<t_{r}$ where $t_{r}$ is the release time of the flow $f_{k}$.
*2.*  $\forall t\in [t_{0},t_{r}]$, at the time t, all channels are occupied by higher-priority flows or there exists node interference between the scheduled flows and the flow $f_{k}$.
*3.*  $\forall t<t_{0},\exists t^{\prime }\in \left[t,t_{0}\right]$, at the time $t^{\prime }$, there is no node interference, and at least one channel is occupied by lower-priority flows or idle.

If there is no $t_{0}$ that satisfies all conditions, then $t_{0}=t_{r}$.

**Throrem 1.**  *The workload bounds can be computed with:*

(2) $$W_{k}^{NC}\left(f_{i},x\right)=W_{k}^{CI}\left(f_{i},x\right)=\left\lfloor \frac{x}{T_{i}}\right\rfloor \cdot c_{i}+\min\left\{x\;\mathrm{mod}\;T_{i},c_{i}\right\},$$

*where $c_{i}$ is the number of hops in the path $\pi_{i}$, i.e., $c_{i}=\left|\pi_{i}\right|$*

**Figure 3:**
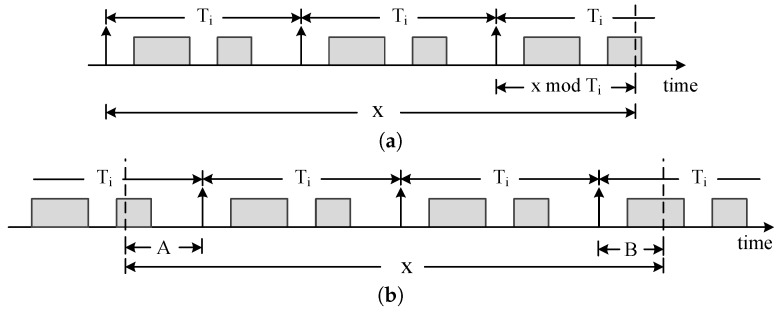
Illustration of Theorem 1. (**a**) $W_{k}^{NC}\left(f_{i},x\right)$; (**b**) $W_{k}^{CI}\left(f_{i},x\right)$.

**Proof of Theorem 1.**  The computation of the non-carry-in workload $W_{k}^{NC}\left(f_{i},x\right)$ is shown in Figure 3a. There are $\left\lfloor \frac{x}{T_{i}}\right\rfloor$ complete periods and a scheduling window $\left(x\;\mathrm{mod}\;T_{i}\right)$. In the scheduling window, at most $c_{i}$ workloads exist. Therefore, the expression of the non-carry-in workload is shown as Equation (2).

In the following, we compute $W_{k}^{CI}$ as shown in Figure 3b. The notations *A* and *B* denote the two incomplete periods, respectively. We know that $A<T_{i}$, $B<T_{i}$ and $A+B=\left(x\;\mathrm{mod}\;T_{i}\right)\;\text{or}\;\left(x\;\;\mathrm{mod}\;T_{i}+T_{i}\right)$. We discuss the two cases as follows.

**Figure 4:**
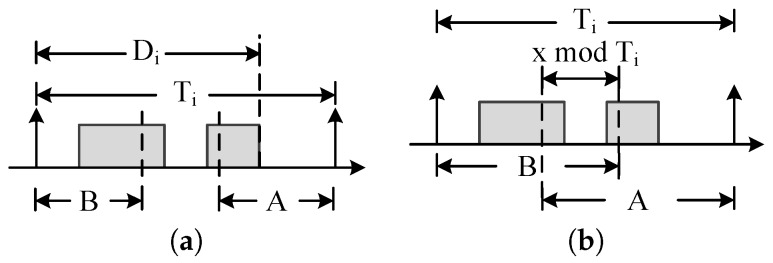
Computation of $W_{k}^{CI}$. (**a**) $A+B=x\;\mathrm{mod}\;T_{i}$; (**b**) $A+B=x\;\mathrm{mod}\;T_{i}+T_{i}$.

Case 1: $A+B=x\;\mathrm{mod}\;T_{i}$. We draw out the windows *A* and *B* in Figure 4a. We consider four different value ranges of the windows *A* and *B* as shown in Table 1, in which if $A\geq T_{i}-D_{i}$ and $B\geq D_{i}$; it is Case 2. If $A<T_{i}-D_{i}$, then there is no workload in *A*. If $B\geq D_{i}$, then all execution time $c_{i}$ must be the available workload. In this case, the workload can also be expressed as $\min\{B,c_{i}\}$. Therefore, only if $A<T_{i}-D_{i}$, the workload is $\min\{B,c_{i}\}$. If $A\geq T_{i}-D_{i}$ and $B<D_{i}$, the time interval $T_{i}-D_{i}$ does not contain any workload. Therefore, the available window A+B is equal to $\left(x\;\mathrm{mod}\;T_{i}\right)-\left(T_{i}-D_{i}\right)$.

In Case 1, we can get that the total workload is:

(3) $$\left\lfloor \frac{x}{T_{i}}\right\rfloor \cdot c_{i}+C1$$

The notation C1 denotes the workload in the incomplete period as shown in Table 1. It is equal to $\min\{B,c_{i}\}$ or $\min\{x\;\mathrm{mod}\;T_{i}-\left(T_{i}-D_{i}\right),c_{i}\}$.

Case 2: $A+B=x\;\mathrm{mod}\;T_{i}+T_{i}$, which is shown in Figure 4b. In this case, there are $\left\lfloor \frac{x-T_{i}}{T_{i}}\right\rfloor$ complete periods. In the windows *A* and *B*, at most $c_{i}+\min\left\{x\;\mathrm{mod}\;T_{i},c_{i}\right\}$ workloads exist. Therefore, the workload of Case 2 is:

$$\left\lfloor \frac{x-T_{i}}{T_{i}}\right\rfloor \cdot c_{i}+c_{i}+\min\left\{x\;\mathrm{mod}\;T_{i},c_{i}\right\}$$

(4) $$\Rightarrow \left\lfloor \frac{x}{T_{i}}\right\rfloor \cdot c_{i}+\min\left\{x\;\mathrm{mod}\;T_{i},c_{i}\right\}$$

Comparing with Equations (3) and (4), the upper bound of workload is Equation (4). Since $\left(x\;\;\mathrm{mod}\;T_{i}\right)$ is not less than *B* and $\left(x\;\mathrm{mod}\;T_{i}\right)-\left(T_{i}-D_{i}\right)$, Equation (4) is the same as Equation (2). The theorem holds. ☐

Due to the two types of workload having the same computing formula, we do not distinguish them in the following and use $W_{k}\left(f_{i},x\right)$ to denote them. Based on the workload bound, the interference of the flow $f_{i}$ to the flow $f_{k}$ is:

$$I_{k}\left(f_{i},x\right)=\min\left\{\max\left\{W_{k}\left(f_{i},x\right),0\right\},x-c_{k}+1\right\}$$

Thus, the total interference suffered by the flow $f_{k}$ is:

$$\Omega_{k}^{total}\left(x,\bar{P}\left(f_{k}\right)\right)=\sum_{\forall f_{i}\in \bar{P}\left(f_{k}\right)}I_{k}\left(f_{i},x\right)$$

#### 6.1.2. Worst Case Delay in Single-Criticality Networks

$\Omega_{k}^{n}\left(x,\bar{P}\left(f_{k}\right)\right)$ and $\Omega_{k}^{s}\left(x,\bar{P}\left(f_{k}\right)\right)$ denote node interference and scheduling interference suffered by the flow $f_{k}$ in the level-*k* busy period. If there exists a node interference at a time slot, the flow $f_{k}$ cannot be transmitted at this time slot, no matter how many channels are idle, i.e., the flow $f_{k}$ is delayed one time slot due to the node interference. However, only when *M* transmissions are scheduled at a time slot, the flow $f_{k}$ suffers scheduling interference and is delayed for one time slot. In the worst case, all of the node interference and scheduling interference will introduce delay to the flow $f_{k}$. Therefore, the worst case delay is:

(5) $$\Omega_{k}^{n}\left(x,\bar{P}\left(f_{k}\right)\right)+\left\lfloor \frac{\Omega_{k}^{s}\left(x,\bar{P}\left(f_{k}\right)\right)}{M}\right\rfloor +c_{k}$$

From Equation (5), we know that node interference introduces more delay. Since the sum of node interference and scheduling interference is $\Omega_{k}^{total}\left(x,\bar{P}\left(f_{k}\right)\right)$, so when as much as possible node interference occurs, the end-to-end delay is the worst case.

The upper bound of node interference introduced by *h* consecutive hops of the flow $f_{i}$ to the flow $f_{k}$ is computed as:

$$R_{k,i}\left(h\right)=\max_{\forall a\in [1,c_{i}-h]}\left\{\right|\left\{\tau_{iy}|\forall \tau_{iy},y\in \left[a,a+h\right],\exists \tau_{kz}\;\text{such}\;\text{that}\;\tau_{iy}\cap \tau_{kz}\neq \emptyset \right\}\left|\right\}$$

Thus, the workload introduced by transmissions that have node interference is:

$$W_{k}^{n}\left(f_{i},x\right)=\left\lfloor \frac{x}{T_{i}}\right\rfloor \cdot R_{k,i}\left(c_{i}\right)+R_{k,i}\left(\min\left\{x\;\mathrm{mod}\;T_{i},c_{i}\right\}\right)$$

Then,

$$I_{k}^{n}\left(f_{i},x\right)=\min\left\{\max\left\{W_{k}^{n}\left(f_{i},x\right),0\right\},x-c_{k}+1\right\}$$

and:

$$\Omega_{k}^{n}\left(x,\bar{P}\left(f_{k}\right)\right)=\sum_{\forall f_{i}\in \bar{P}\left(f_{k}\right)}I_{k}^{n}\left(f_{i},x\right)$$

Then, we can get that the worst case delay of the flow $f_{k}$ in the single-criticality network is:

$$D_{k}=\Omega_{k}^{n}\left(x,\bar{P}\left(f_{k}\right)\right)+\left\lfloor \frac{\Omega_{k}^{total}\left(x,\bar{P}\left(f_{k}\right)\right)-\Omega_{k}^{n}\left(x,\bar{P}\left(f_{k}\right)\right)}{M}\right\rfloor +c_{k}$$

From the definition of the level-*k* busy period, we know that the length *x* is the upper bound of the delay $D_{k}$ (shown in Theorem 2).

**Theorem 2.**  *For the flow $f_{k}$ and the level-k busy period, the following holds:*

$$x\geq D_{k}$$

**Figure 5:**
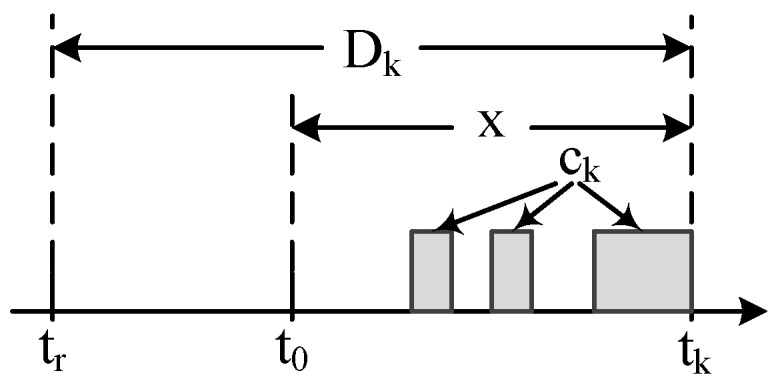
Illustration of Theorem 2.

**Proof of Theorem 2.**  We assume by contradiction that $x<D_{k}$. From the definition of the level-*k* busy period (Definition 2), we know that the finish times of the busy period and the flow $f_{k}$ are the same, and $t_{0}$ must be less than (the first condition) or equal to $t_{r}$ (when $t_{0}$ does not satisfy at lest one condition). If $x<D_{k}$, then $t_{r}<t_{0}$, as shown in Figure 5. It is not consistent with the definition. The above assumption does not hold.

According to Theorem 2, the solution of Equation (6) is the upper bound of end-to-end delay $D_{k}$.

(6) $$x=\Omega_{k}^{n}\left(x,\bar{P}\left(f_{k}\right)\right)+\left\lfloor \frac{\Omega_{k}^{total}\left(x,\bar{P}\left(f_{k}\right)\right)-\Omega_{k}^{n}\left(x,\bar{P}\left(f_{k}\right)\right)}{M}\right\rfloor +c_{k}$$

Equation (6) can be solved by the iterative fixed point search [39]. The iterative calculation of *x* starts with $x=c_{k}$; until the value of *x* does not change.

### 6.2. Mixed Criticality Scheduling Analysis

In dual-criticality networks, there are three types of worst case delay.

(1) $D_{k}^{L}$: the worst case end-to-end delay of the L-crit flow.
(2) $D_{k}^{HL}$: the worst case end-to-end delay of the H-crit flow with the L-crit parameter.
(3) $D_{k}^{H}$: the worst case end-to-end delay of the H-crit flow with the H-crit parameter.

We use D(x,Q,c) to denote $\Omega_{k}^{n}\left(x,Q\right)+\left\lfloor \frac{\Omega_{k}^{total}\left(x,Q\right)-\Omega_{k}^{n}\left(x,Q\right)}{M}\right\rfloor +c$. The methods of computing these types of delay are similar. The only difference is that the higher-priority flows they suffered are different, i.e., their interference sets *Q* are different. H-crit flows have multiple paths. These paths suffer different interference and cause different delays. Therefore, we use sub-flows $f_{k}^{*}$, $f_{k}^{\prime }$ and $f_{k}^{\prime \prime }$ to distinguish them.

If there are H-crit flows with H-crit parameters in networks, L-crit flows can be discarded. Therefore, when we compute the delay $D_{k}^{L}$, all flows have L-crit parameters. Thus, $D_{k}^{L}=D\left(x,Q_{k}^{L},c_{k}^{*}\right)$, where $Q_{k}^{L}=\left\{f_{i}^{*}|\forall f_{i}^{*},T_{i}\left(L\right)<T_{k}\left(L\right)\right\}$ and $c_{k}^{*}=\left|\pi_{k}^{*}\right|$.

Similarly, for H-crit flows with L-crit parameters, the interference set is $Q_{k}^{HL}=\left\{f_{i}^{\prime },f_{i}^{\prime \prime }|\forall f_{i}^{\prime },\forall f_{i}^{\prime \prime },\chi_{i}=H,T_{i}\left(H\right)<T_{k}\left(L\right)\right\}\cup \left\{f_{i}^{*}|\forall f_{i}^{*},T_{i}\left(L\right)<T_{k}\left(L\right)\right\}$. Thus, $D_{k}^{HL}=D\left(x,Q_{k}^{HL},c_{k}^{*}\right)$, where $c_{k}^{*}=\left|\pi_{k}^{*}\right|$.

An H-crit flow with its H-crit parameter suffers the interference from H-crit flows with H-crit parameters. The H-crit flow has two sub-flows $f_{k}^{\prime }$ and $f_{k}^{\prime \prime }$. For these sub-flows, their interference set is $Q_{k}^{H}=\left\{f_{i}^{\prime },f_{i}^{\prime \prime }|\forall f_{i}^{\prime },\forall f_{i}^{\prime \prime },\chi_{i}=H,T_{i}\left(H\right)<T_{k}\left(H\right)\right\}\cup \left\{f_{i}^{*}|\forall f_{i}^{*},\chi_{i}=H,T_{i}\left(L\right)<T_{k}\left(H\right)\right\}$ and $c_{k}^{\prime }=\left|\pi_{k}^{\prime }\right|$, $c_{k}^{\prime \prime }=\left|\pi_{k}^{\prime \prime }\right|$. Thus, $D_{k}^{\prime H}=D\left(x,Q_{k}^{H},c_{k}^{\prime }\right)$ and $D_{k}^{\prime \prime H}=D\left(x,Q_{k}^{H},c_{k}^{\prime \prime }\right)$, and then, $D_{k}^{H}=\max\left\{D_{k}^{\prime H},D_{k}^{\prime \prime H}\right\}$.

According to the above delays, the schedulability test is as follows. For the L-crit flow $f_{k}$, if $D_{k}^{L}\leq T_{k}\left(L\right)$, it is schedulable; otherwise, unschedulable. For the H-crit flow $f_{k}$, if $D_{k}^{HL}\leq T_{k}\left(L\right)$ and $D_{k}^{H}\leq T_{k}\left(H\right)$, it is schedulable; otherwise, unschedulable. If all flows in a flow set are schedulable, the set is schedulable.

## 7. Evaluation

In this section, we conduct experiments to evaluate the performance of our proposed methods.

### 7.1. Scheduling Algorithm

We consider three comparison methods: (1) SMT uses the Z3 solver [35], which is a high-performance solver being developed at Microsoft Research and whose solution has been regarded as an excellent standard, to solve our SMT specification (Section 4); (2) noStealRM applies the RM priority and does not allow slots to be stolen; (3) StealCM allows slots to be stolen and applies the criticality monotonic priority. Our method is StealRM. The performance metric we used is the schedulable ratio, which is defined as the percentage of flow sets for which a scheduling algorithm can find a schedulable solution.

We randomly generate a number of test cases to evaluate these methods. For each test case, the number of channels *M* and the number of nodes |N| are given. According to the suggestion in the work [40], these nodes are placed randomly in the square area *A*, and $A=\frac{\left|N\right|d^{2}\sqrt{27}}{2\pi }$, where the transmitting range *d* is 40 m. Except the gateway, each node has a data flow from itself to the gateway or vice versa. There are two necessary schedulability conditions for flow sets: (1) the network utilization *U* is not larger than one; (2) the utilization of each node is not larger than one. If a flow set does not satisfy the two conditions, it cannot be scheduled. Thus, in order to make flow sets available, we specify the network utilization U(U<1) and use the method UUniFast [41] to assign the utilization $u_{i}$ for each flow, where $U=\sum_{\forall f_{i}\in F}u_{i}$. Then, if the flow set can satisfy the condition (2), it is an available flow set. Otherwise, discard it, and repeat the process until an available set is found. The period of each flow can be obtained according to $T_{i}=\frac{c_{i}}{u_{i}}$. The high-crit probability of the flows is controlled by the parameter *ρ*. Routing paths are selected randomly.

**Figure 6:**
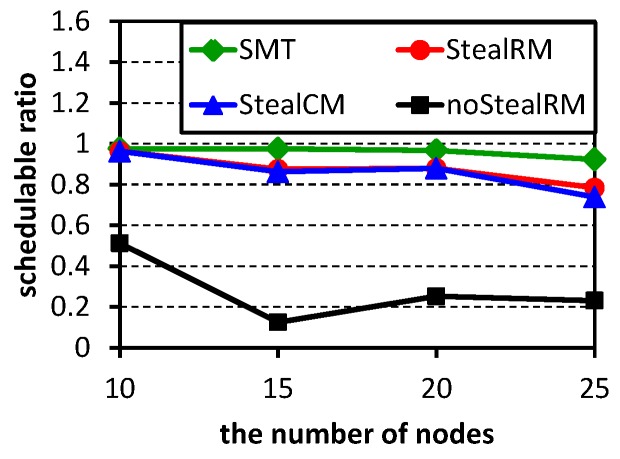
Schedulability comparison among all methods. SMT, satisfiability modulo theories; StealRM, slot-stealing scheduling algorithm based on rate-monotonic; StealCM, slot-stealing scheduling algorithm based on criticality monotonic.

**Figure 7:**
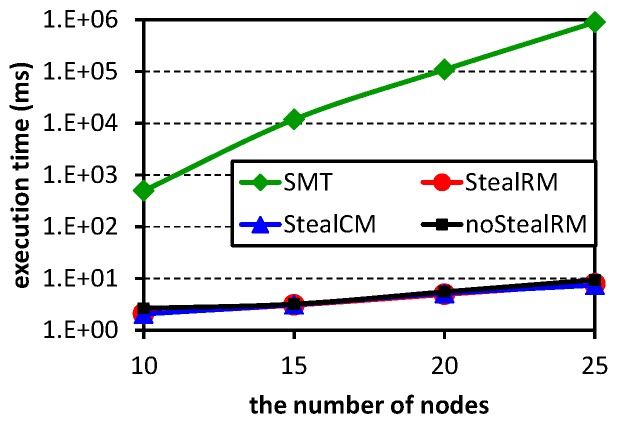
Execution time comparison among all methods.

In order to make test cases solvable by the Z3 solver, the parameters are set as ρ=0.3, M=2 and U=0.8. For each configuration, 100 test cases are checked using the four algorithms. Figure 6 shows their schedulable ratios. Our algorithm StealRM is close to the result of Z3. In these simple test cases, the method StealCM has similar results with our algorithm StealRM. Figure 7 shows the average execution time of the solvable test cases in Figure 6. When the number of nodes is 25, the execution time of the method SMT is about 16.5 min. We also use the method SMT to solve the network with 30 nodes, but cannot get the result within 3 h. Except the method SMT, the execution time of other methods is not more than 10 milliseconds. Therefore, from the perspective of execution time, heuristic algorithms are significantly more efficient than the method SMT.

**Figure 8:**
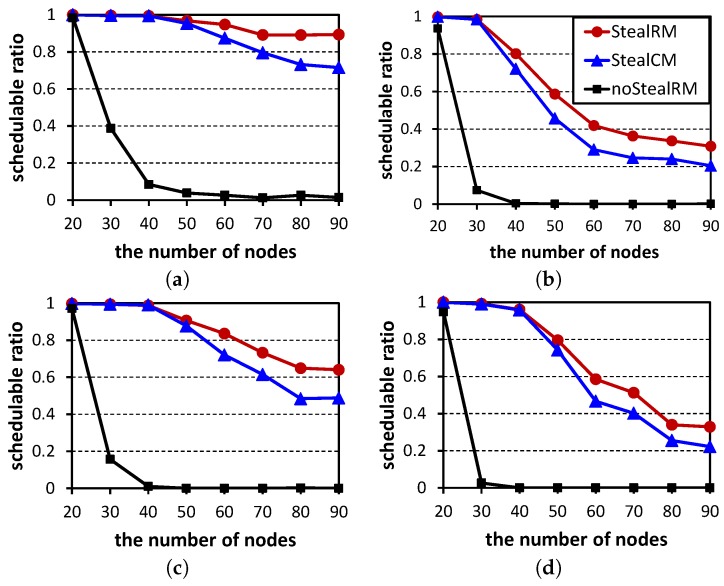
Schedulability comparison among StealRM, StealCM and noStealRM. (**a**) M=6,U=0.5, ρ=0.3; (**b**) M=6,U=0.5,ρ=0.4; (**c**) M=6,U=0.6,ρ=0.3; (**d**) M=9,U=0.5,ρ=0.3.

Since the execution time of the method SMT is too long, the following experiments do not contain it. Figure 8 shows the schedulable ratios of the three scheduling algorithms. For each point in the figure, 500 test cases are randomly generated. From the figure, we can know that our algorithm StealRM has the highest schedulable ratio no matter with which parameters, while the algorithm noStealRM has the worst result. Therefore, the stealing mechanism can significantly improve the algorithm’s performance. Our algorithm StealRM has better performance than the algorithm StealCM, especially when the node numbers are higher. This demonstrates that: (1) the priority should correspond to the urgency, but not the importance, while the stealing mechanism reflects the importance; (2) the urgency and the importance have to be distinguished, except in very small networks. Comparing among these subfigures, we observe that schedulable ratios decrease with the increases of *ρ*, |N|, *U* and *M*. The reasons are as follows. An H-crit flow can be regarded as two L-crit flows. Thus, a larger value of the parameter *ρ* leads to more flows, which are hard to schedule. A test case contains |N|-1 flows. Likewise, the larger |N| makes scheduling hard. The network utilization *U* corresponds to the network workload. Heavy workloads lead to scheduling failures. Note that comparing with Figure 8a, Figure 8d has three additional channels, but its schedulable ratios decrease. Because the two subfigures generate test cases according to the respective numbers of channels. Their test cases are different. Although the number of channels increases, the utilization is not changed. When the utilization *U* is constant, with the increase of the number of channels *M*, the packets that need to be transmitted increase. The increased packets will introduce more interference, which have a negative impact on the scheduling performance. Therefore, Figure 8d has a lower schedulable ratio than Figure 8a.

**Figure 9:**
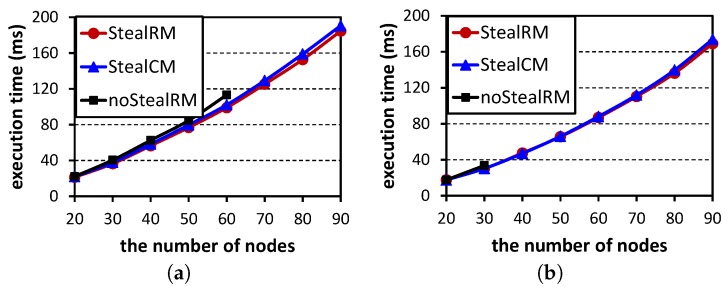
Average execution time. (**a**) M=6,U=0.5,ρ=0.3; (**b**) M=9,U=0.5,ρ=0.3.

Figure 9 shows the average execution time of Figure 8. As the results are similar, we only show two subfigures for Figure 8a,d. Comparing with our algorithm StealRM, the algorithms StealCM and noStealRM need more time to find feasible solutions. Therefore, their execution time slightly increases. From the figure, we know that our algorithm StealRM does not introduce extra time cost. For the three algorithms, the execution time increases with the increases of the number of nodes, since more data flows need to be scheduled.

### 7.2. Analyzing Method

**Figure 10:**
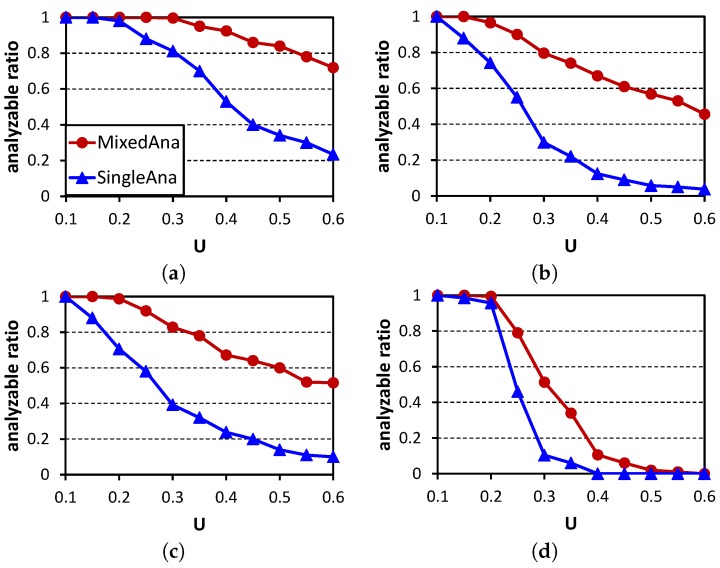
Schedulability comparison among analyzing algorithms. (**a**) |N|=20,M=6,ρ=0.1; (**b**) |N|=20,M=6,ρ=0.3; (**c**) |N|=20,M=9,ρ=0.1; (**d**) |N|=60,M=6,ρ=0.1. MixedAna, mixed criticality analysis.

**Figure 11:**
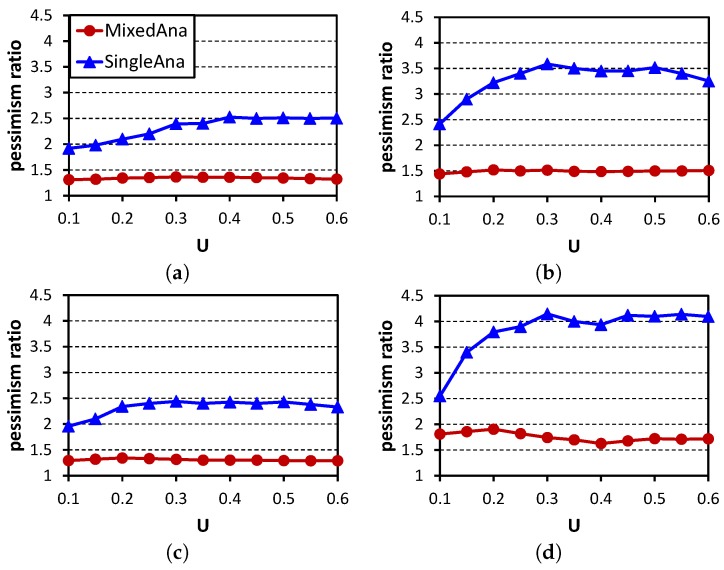
Delay comparison with StealRM being used as the baseline. (**a**) |N|=20,M=6,ρ=0.1; (**b**) |N|=20,M=6,ρ=0.3; (**c**) |N|=20,M=9,ρ=0.1; (**d**) |N|=60,M=6,ρ=0.1.

The comparison method is SingleAna, in which flow sets are tested using the single-criticality analysis. Our mixed criticality analysis method is MixedAna. The performance metrics are the analyzable ratio (the percentage of flow sets which are tested as schedulable by an analyzing method) and the pessimism ratio (the proportion of analyzed delay to the delay observed in StealRM). Figure 10 shows the comparison of the analyzable ratios. For each point, 500 test cases are analyzed. Our method MixedAna outperforms SingleAna. The analyzable ratios decrease with the increases of these parameters. The reasons are similar to those in Figure 8. The increases of *U* and *M* lead to more packets, and the increases of |N| and *ρ* lead to more flows. These will cause more interference. Thus, the analysis introduces more pessimism, and the analyzable ratios decrease. Figure 11 shows the pessimism ratios of experiments in Figure 10. The pessimism ratios of MixedAna are less than two, while the pessimism ratios of SingleAna are all larger than two. This is because the interference that does not exist between H-crit and L-crit flows is eliminated in MixedAna.

## 8. Conclusions

Multiple criticality levels coexist in advanced industrial applications. They share the network resource, but their requirements for the real-time performance and reliability are different. In this paper, we propose a scheduling algorithm to guarantee their different requirements and then analyze the schedulability for this scheduling algorithm. Simulation results show that our scheduling algorithm and analysis have more performance than existing ones. In the future work, we will propose a routing algorithm for mixed criticality WSNs to enhance the reliability and design a network deployment method and a parameter adjustment method to improve the schedulability. Finally, we will implement these algorithms in a real network.

## Figures and Tables

**Table 1:** The workload in the incomplete period under different value ranges of *A* and *B*.

Workload	A < T${}_{i}$ – D ${}_{i}$	A ≥ T${}_{i}$ – D ${}_{i}$
$B\geq D_{i}$	$c_{i}$	**Case 2**
$B<D_{i}$	$\min\{B,c_{i}\}$	$\min\{x\;\mathrm{mod}\;T_{i}-\left(T_{i}-D_{i}\right),c_{i}\}$
**C1**	$\min\{B,c_{i}\}$	$\min\{x\;\mathrm{mod}\;T_{i}-\left(T_{i}-D_{i}\right),c_{i}\}$

## References

[1] Liang W., Zhang X., Xiao Y., Wang F., Zeng P., Yu H. (2011). Survey and experiments of WIA–PA specification of industrial wireless network. Wirel. Commun. Mob. Comput..

[2] Std, IEC (2009). Industrial Communication Networks—Wireless Communication Network and Communication Profiles–WirelessHART.

[3] Nobre M., Silva I., Guedes L.A. (2015). Routing and scheduling algorithms for wirelessHART networks: A survey. Sensors.

[4] Quang P.T.A., Kim D.S. (2014). Throughput-aware routing for industrial sensor networks: Application to ISA100. 11a. IEEE Trans. Ind. Inf..

[5] Saifullah A., Xu Y., Lu C., Chen Y. Real-time scheduling for WirelessHART networks. Proceedings of the 2010 IEEE 31st Real-Time Systems Symposium (RTSS).

[6] Saifullah A., Xu Y., Lu C., Chen Y. End-to-end delay analysis for fixed priority scheduling in WirelessHART networks. Proceedings of the 2011 17th IEEE Real-Time and Embedded Technology and Applications Symposium (RTAS).

[7] Zhang H., Cheng P., Shi L., Chen J. (2015). Optimal DoS attack scheduling in wireless networked control system. IEEE Trans. Control Syst. Technol..

[8] Cheng P., Qi Y., Xin K., Chen J., Xie L. (2015). Energy-efficient data forwarding for state estimation in multi-hop wireless sensor networks. IEEE Trans. Autom. Control.

[9] Liu X., Hou K.M., de Vaulx C., Shi H., Gholami K.E. (2014). MIROS: A hybrid real-time energy-efficient operating system for the resource-constrained wireless sensor nodes. Sensors.

[10] Yu S., Zhang X., Liang W. (2016). Concurrent transmission performance modeling of wireless multimedia sensor network and its experimental evaluation. Inf. Control.

[11] Baruah S., Bonifaci V., D’Angelo G., Li H., Marchetti-Spaccamela A., Megow N., Stougie L. (2012). Scheduling real-time mixed-criticality jobs. IEEE Trans. Comput..

[12] Burns A., Davis R. (2015). Mixed criticality systems-a review. Tech. Rep..

[13] Zhang H., Soldati P., Johansson M. Optimal link scheduling and channel assignment for convergecast in linear WirelessHART networks. Proceedings of the WiOPT 2009 7th International Symposium on Modeling and Optimization in Mobile, Ad Hoc, and Wireless Networks.

[14] Chipara O., Lu C., Roman G.C. (2013). Real-time query scheduling for wireless sensor networks. IEEE Trans. Comput..

[15] Huang H.M., Gill C., Lu C. (2014). Implementation and evaluation of mixed-criticality scheduling approaches for sporadic tasks. ACM Trans. Embed. Comput. Syst..

[16] Vestal S. Preemptive scheduling of multi-criticality systems with varying degrees of execution time assurance. Proceedings of the RTSS 2007 28th IEEE International Real-Time Systems Symposium.

[17] Burns A., Fleming T., Baruah S. Cyclic executives, multi-core platforms and mixed criticality applications. Proceedings of the 2015 27th Euromicro Conference on Real-Time Systems (ECRTS).

[18] Lee J., Phan K.M., Gu X., Lee J., Easwaran A., Shin I., Lee I. Mc-fluid: Fluid model-based mixed-criticality scheduling on multiprocessors. Proceedings of the 2014 IEEE Real-Time Systems Symposium.

[19] Burns A., Davis R.I. Mixed criticality on controller area network. Proceedings of the 2013 25th Euromicro Conference on Real-Time Systems (ECRTS).

[20] Cros O., Fauberteau F., George L., Li X. (2014). Mixed-criticality over switched ethernet networks. Ada User J. Proc. Workshop Mixed Crit. Ind. Syst..

[21] Addisu A., George L., Sciandra V., Agueh M. Mixed criticality scheduling applied to jpeg2000 video streaming over wireless multimedia sensor networks. Proceedings of the 2013 19th International Conferecne on Embedded and Real-Time Computing Systems and Applications (RTCSA) Workshop on Mixed Criticality Systems (WMC).

[22] Shen W., Zhang T., Barac F., Gidlund M. (2014). PriorityMAC: A priority-enhanced MAC protocol for critical traffic in industrial wireless sensor and actuator networks. IEEE Trans. Ind. Inf..

[23] Hussain S.W., Khan T., Zaidi S.H. Latency and energy efficient MAC (LEEMAC) Protocol for event critical applications in WSNs. Proceedings of the CTS 2006 International Symposium on Collaborative Technologies and Systems.

[24] Soldati P., Zhang H., Johansson M. Deadline-constrained transmission scheduling and data evacuation in WirelessHART networks. Proceedings of the 2009 European Control Conference.

[25] Zhang H., Osterlind F., Soldati P., Voigt T., Johansson M. (2015). Time-optimal convergecast with separated packet copying: Scheduling policies and performance. IEEE Trans. Veh. Technol..

[26] Saifullah A., Xu Y., Lu C., Chen Y. Priority assignment for real-time flows in WirelessHART networks. Proceedings of the 2011 23rd Euromicro Conference on Real-Time Systems (ECRTS).

[27] Baruah S., Vestal S. Schedulability analysis of sporadic tasks with multiple criticality specifications. Proceedings of the ECRTS ’08 Euromicro Conference on Real-Time Systems.

[28] Burns A., Harbin J., Indrusiak L.S. A wormhole noc protocol for mixed criticality systems. Proceedings of the IEEE Real-Time Systems Symposium (RTSS).

[29] Tobuschat S., Axer P., Ernst R., Diemer J. IDAMC: A NoC for mixed criticality systems. Proceedings of the 2013 IEEE 19th International Conference on Embedded and Real-Time Computing Systems and Applications (RTCSA).

[30] Carvajal G., Fischmeister S. An open platform for mixed-criticality real-time ethernet. Proceedings of the IEEE Design, Automation & Test in Europe Conference & Exhibition.

[31] Koubâa A., Alves M., Tovar E., Cunha A. (2008). An implicit GTS allocation mechanism in IEEE 802.15. 4 for time-sensitive wireless sensor networks: Theory and practice. Real-Time Syst..

[32] Zhan Y., Xia Y., Anwar M. (2016). GTS size adaptation algorithm for IEEE 802.15. 4 wireless networks. Ad Hoc Netw..

[33] Jin X., Wang J., Zeng P. (2015). End-to-end delay analysis for mixed-criticality wirelesshart networks. IEEE/CAA J. Autom. Sin..

[34] Li B., Nie L., Wu C., Gonzalez H., Lu C. Incorporating emergency alarms in reliable wireless process control. Proceedings of the ACM/IEEE Sixth International Conference on Cyber-Physical Systems.

[35] De Moura L., Bjørner N. (2008). Z3: An efficient SMT solver. Tools and Algorithms for the Construction and Analysis of Systems.

[36] Dutertre B., de Moura L. System description: Yices 1.0. Proceedings of the Satisfiability Modulo Theories Competition.

[37] Pellizzoni R., Paryab N., Yoon M.K., Bak S., Mohan S., Bobba R.B. A generalized model for preventing information leakage in hard real-time systems. Proceedings of the IEEE Real-Time and Embedded Technology and Applications Symposium (RTAS).

[38] Guan N., Stigge M., Yi W., Yu G. New response time bounds for fixed priority multiprocessor scheduling. Proceedings of the 30th IEEE Real-Time Systems Symposium.

[39] Joseph M., Pandya P. (1986). Finding response times in a real-time system. Comp. J..

[40] Camilo T., Silva J.S., Rodrigues A., Boavida F. (2007). Gensen: A topology generator for real wireless sensor networks deployment. Software Technologies for Embedded and Ubiquitous Systems.

[41] Bini E., Buttazzo G.C. (2005). Measuring the performance of schedulability tests. Real-Time Syst..

